# miR-939-3p induces sarcoma proliferation and poor prognosis via suppressing BATF2

**DOI:** 10.3389/fonc.2024.1346531

**Published:** 2024-02-14

**Authors:** Wanwen Xu, Yinghui Huang, Zengjie Lei, Jie Zhou

**Affiliations:** ^1^ Wuhan Third Hospital (Tongren Hospital of Wuhan University), Wuhan, Hubei, China; ^2^ Department of Nephrology, The Key Laboratory for the Prevention and Treatment of Chronic Kidney Disease of Chongqing, Chongqing Clinical Research Center of Kidney and Urology Diseases, Xinqiao Hospital, Army Medical University, Chongqing, China; ^3^ Department of Medical Oncology, Affiliated Jinling Hospital, Medical School of Nanjing University, Nanjing, China; ^4^ Department of Oncology and Southwest Cancer Center, Southwest Hospital, Army Medical University (Third Military Medical University), Chongqing, China

**Keywords:** BATF2, miR-939-3p, sarcoma, prognosis, therapeutic target

## Abstract

**Background:**

Sarcoma is a rare and aggressive malignancy with poor prognosis, in which oncogene activation and tumor suppressor inactivation are involved. Accumulated studies suggested basic leucine zipper transcription factor ATF-like 2 (BATF2) as a candidate tumor suppressor, but its specific role and mechanism in sarcoma remain unclear.

**Methods:**

The expression levels of BATF2 and miR-939-3p were evaluated by using human sarcoma samples, cell lines and xenograft mouse models. Bioinformatics analysis, qPCR, Western blot, cell proliferation assay, overexpression plasmid construction, point mutation and dual luciferase reporter assay were utilized to investigate the role and mechanism of miR-939-3p in sarcoma.

**Results:**

In this study, we demonstrated that the expression of BATF2 was downregulated in human sarcoma tissues and cell lines. The downregulation of BATF2 was negatively associated with the prognosis of sarcoma patients. Subsequent bioinformatic prediction and experimental validations showed that BATF2 expression was reduced by microRNA (miR)-939-3p mimic and increased by miR-939-3p inhibitor. Additionally, miR-939-3p was upregulated in sarcoma tissues and cells, correlating with a poor prognosis of sarcoma patients. Moreover, miR-939-3p overexpression suppressed sarcoma cell proliferation, which was significantly attenuated by the restoration of BATF2, while siRNA-mediated knockdown of BATF2 aggravated the miR-939-3p-induced promotion of sarcoma cell proliferation. Further computational algorithms and dual-luciferase reporter assays demonstrated that miR-939-3p repressed BATF2 expression via directly binding to its 3’ untranslated region (3’ UTR).

**Conclusion:**

Collectively, these findings identified miR-939-3p as a novel regulator of BATF2, as well as a prognostic biomarker in sarcoma, and revealed that suppressing miR-939-3p or inducing BATF2 expression may serve as a promising therapeutic strategy against sarcoma.

## 1 Introduction

Sarcomas are rare cancers predominantly derived from embryonic mesoderm, accounting for approximately 1% of human malignancies, which primarily affect younger patients and are difficult to treat (1–3). The histologic and molecular diversity, coupled with limited access to specialized sarcoma centers, often leads to erroneous therapeutic plans (4). Currently, localized sarcomas are typically treated with surgery, and chemotherapy or targeted therapy is employed for metastatic disease (2), while immunotherapy for sarcoma remains largely investigational (2, 4, 5). Despite numerous clinical trials exploring various chemotherapy regimens, the progression-free survival rates range from 3 to 7 months, with an overall survival (OS) of 12 to 18 months (6, 7). Therefore, gaining a comprehensive insight into the mechanism underlying sarcoma tumorigenesis and progression is crucial to develop effective therapeutic approaches against this disease.

Among the sarcoma-related risk factors, the activation of oncogenes and silencing of tumor suppressor genes play vital roles during sarcoma tumorigenesis (2, 8). Recently, we and others have identified basic leucine zipper transcription factor ATF-like 2 (BATF2) as a tumor suppressor (9–18). BATF2 is mainly expressed in normal cells but not in the corresponding tumor cells, while the restoration of BATF2 inhibits cancer cell proliferation, invasion and metastasis (13, 14), highlighting its potential as a therapeutic target in cancer. Further investigation into BATF2 dysfunction revealed no mutations in the exons of the *BATF2* gene in hepatocellular carcinoma (16). Consequently, multiple studies, including our own, focused on elucidating the upstream transcription factors, N6-methyladenosine modification and nuclear-cytoplasmic translocation of BATF2 to understand gene expression regulation (9, 10, 13–15, 18–20). However, these findings have still not clarified the cause of BATF2 downregulation. Further, its role and underlying mechanisms in sarcoma remain unknown.

MicroRNAs (miRs) are small non-coding RNAs, possessing approximately 22 nucleotides, which can regulate gene expression through binding to the 3’ untranslated region (3’ UTR) of its target mRNAs, ultimately contributing to mRNA cleavage and translational inhibition (21, 22). As post-transcriptional regulators, miRNAs have been implicated in modulating various malignant behaviors, including cancer cell proliferation, apoptosis, motility, adhesion and drug resistance (22). Many miRNAs are directly or indirectly associated with oncogenes and can function as either tumor suppressors or oncomiRs (23). During sarcoma initiation and progression, changes in the expression patterns of certain miRNAs significantly impact sarcoma cell proliferation, invasion, and apoptosis (8). However, the precise molecular mechanism by which miRNAs regulate BATF2 to enhance sarcoma growth remains elusive.

In this study, we demonstrated a downregulation of BATF2 in sarcoma, which was positively correlated with prognosis. *In vitro* and *in vivo* experiments revealed that miR-939-3p enhanced sarcoma proliferation through suppressing BATF2 expression via binding to its 3’ UTR. These findings highlight the miR-939-3p/BATF2 axis as a promising therapeutic target for sarcoma patients.

## 2 Materials and methods

### 2.1 Cell culture

The human fibrosarcoma cell line HT-1080, synovial sarcoma cell line SW-982 and human skin fibroblast cell line HSF were obtained from iCell Bioscience (Shanghai, China). The stable transfection of HT-1080 cells was performed as we described (9). Briefly, HT-1080 cells were transfected with lentivirus-packaged recombinant plasmids expressing miR-939-3p or negative control (Tsingke Biotechnology, Shanghai, China), and resistant cells were selected by using Puromycin (Sigma, St. Louis, MO, USA) to establish stable cell lines.

### 2.2 Human samples

Sarcoma, colorectal cancer (CRC) and the corresponding paratumor tissues from 12 patients were separately collected from the People’s Hospital of Xishui County (Zunyi, China). The study was carried out in accordance with the Declaration of Helsinki and approved by the People’s Hospital of Xishui County. Written informed consent has been obtained from all patients.

### 2.3 Animal studies

Four-week-old female nude mice were purchased from the Beijing Huafukang Biotechnology (Beijing, China). The mice were subcutaneously injected with 5 × 10^6^ HT-1080 cells in 0.2 mL PBS per mouse with or without stable miR-939-3p overexpression (n = 5). After 21 days, the xenografts were excised, measured for volume and weight, and further processed for RNA and protein extraction, which was approved by the Animal Welfare and Ethics Committee of Army Medical University.

### 2.4 qPCR

Total RNA of HT-1080 cells and tumor tissues was extracted by using an RNA Purified Total RNA Extraction Kit (Beyotime), and then reversely transcribed into cDNA with RT Master Mix (MedChemExpress, Monmouth Junction, NJ, USA), as we previously described (24). qPCR was performed using a SYBR Green qPCR kit (MedChemExpress). Specific primer sets were provided in **Supplementary Table S1**.

### 2.5 miRNA extraction and qPCR

To quantify miR-939-3p, total RNA, including miRNA, was extracted using the RNAiso Plus reagent (TaKaRa, Shiga, Japan), which was reversely transcribed into cDNA by using a miRNA First Strand cDNA Synthesis kit from Sangon (Shanghai, China). qPCR was performed using a SYBR Green qPCR kit (MedChemExpress). The primer sets were listed in **Supplementary Table S2**.

### 2.6 Western blot

Western blot was performed as we described (24). Briefly, proteins were extracted from sarcoma cells and tissues by using RIPA lysis buffer (Beyotime). Proteins were separated and transferred onto PVDF membranes, which were then incubated with the antibodies against BATF2 (Abcam, Cambridge, MA, USA) or β-actin (Santa Cruz, Dallas, TX, USA), followed by the incubation of secondary antibody. Gray-scale of the bands were quantified by using ImageJ software.

### 2.7 Immunohistochemistry staining

Immunohistochemistry (IHC) staining was performed to detect the expression levels of BATF2 (Abcam) and Ki67 (Santa Cruz) in tumor xenografts as we previously described (9).

### 2.8 Gene expression profiling interactive analysis

GEPIA (25) is a publicly available platform integrating data based on The Cancer Genome Atlas (TCGA) and Genotype-Tissue Expression (GTEx) project. It provides comprehensive pan-cancer analysis of RNA-sequencing data, including gene expression, correlation, and survival analysis.

### 2.9 CancerMIRNome

The correlation between miR-939-3p expression levels and the prognosis of sarcoma patients, as well as the circulating miR-939-3p expression levels, were analyzed by using CancerMIRNome online database (26). CancerMIRNome is publicly available that enables analysis of miRNAs from TCGA projects and circulating miRNome datasets. Data are available from the Gene Expression Omnibus (GSE124158).

### 2.10 miRNA binding sites prediction

Potential miRNAs targeting BATF2 were predicted using miRDB, miRwalk and targetScan software programs. The potential binding sites of miR-939-3p in BATF2 3′ UTR were analyzed by using targetScan online software.

### 2.11 miRNA overexpression and knockdown

MiR-939-3p mimics or inhibitors, and negative controls were purchased from Tsingke Biotechnology, which were listed in **Supplementary Table S3**. HT-1080 cells were harvested 48 h after transfection using Lipofectamine 3000 (Invitrogen).

### 2.12 BATF2 overexpression and knockdown

Mammalian BATF2 overexpression plasmids (pBATF2) and empty plasmid vector pcDNA3.1 (pControl) were purchased from Tsingke Biotechnology for transfection into HT-1080 cells by using Lipofectamine 3000 (Invitrogen). siRNA targeting BATF2 and scramble siRNA were bought from Santa Cruz.

### 2.13 Cell proliferation assay

HT-1080 cells were seeded into a 96-well plate and co-transfected with vector control or BATF2 overexpression plasmids and miR-939-3p mimic or inhibitor. Cell viability was determined by using Cell Counting Kit 8 (CCK-8) (Dojindo, Kumamoto, Japan) after 48 h or a Bromodeoxyuridine (BrdU) ELISA kit (Roche, Burgess Hill, UK) according to the manufacturer’s instructions.

### 2.14 Reporter plasmids construction and point mutation

The human *BATF2* 3’ UTR sequences containing putative binding sites of miR-939-3p were cloned into pGL4 vector (Promega, Madison, WI, USA). The primer sets and restriction enzymes were listed in **Supplementary Table S4**. For point mutation, the binding sites (5′-GCCCAGG-3′) of miR-939-3p in *BATF2* 3′ UTR were mutated into 5′-ATTTCAA-3′ by using a MutanBEST kit (Takara).

### 2.15 Dual luciferase reporter assay

HT-1080 cells were transiently co-transfected with the recombinant plasmids or pAP-1-Luc (BD Biosciences, Oxford, UK) and negative control or miR-939-3p mimic along with Renilla plasmids using Lipofectamine 3000 (Invitrogen) for dual-luciferase reporter gene assay (Promega), as we previously described (27).

### 2.16 Statistical analysis

Data were presented as mean ± SD and analyzed by using Graphpad Prism 8 software. Comparisons were statistically significant at *P* < 0.05 by using unpaired *t*-test or ANOVA.

## 3 Results

### 3.1 Downregulation of BATF2 correlates with poor prognosis of sarcoma

**Figure 1:**
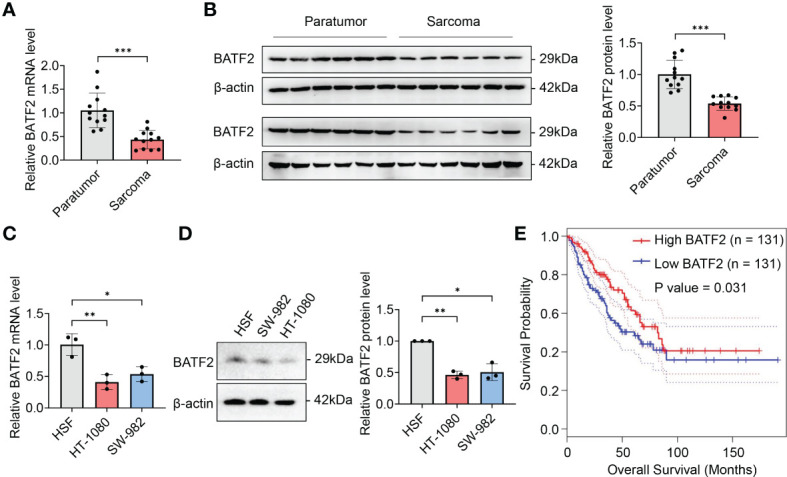
Downregulation of BATF2 correlates with poor prognosis of sarcoma. **(A–D)** qPCR and Western blot analysis of the expression level of BATF2 in soft tissue sarcoma and pericarcinoma **(A, B)** and the human skin fibroblast cell line (HSF), human fibrosarcoma cell line (HT-1080) and synovial sarcoma cell line (SW-982) **(C, D)**. **(E)** Kaplan-Meier estimates of overall survival time based on BATF2 expression levels from 262 CRC patients by using a GEPIA database. Hazard ratio (HR) = 0.65. *P*(HR) = 0.031. *P < 0.05, **P < 0.01, ***P < 0.001.

We first explored the expression levels of BATF2 in human soft tissue sarcoma. It was found that BATF2 mRNA and protein levels were significantly decreased in 12 cases of sarcoma tissues, compared to the corresponding adjacent tissues (**Figures 1A, B**). Besides, BATF2 mRNA and protein expression was also downregulated in human fibrosarcoma cells (HT-1080) and synovial sarcoma cells (SW-982), compared with that in human skin fibroblast cells (HSF) (**Figures 1C, D**). Further, sarcoma patients were divided into two groups according to BATF2 expression levels. Compared with patients with high BATF2 expression (n = 131), patients with low BATF2 expression (n = 131) had obviously worse survival (*P* = 0.031) with a hazard ratio of 0.65 (*P* = 0.031) (**Figure 1E**). These findings indicated that BATF2 was downregulated in sarcoma and negatively associated with the prognosis of sarcoma patients.

### 3.2 miR-939-3p inhibits BATF2 expression in sarcom

**Figure 2:**
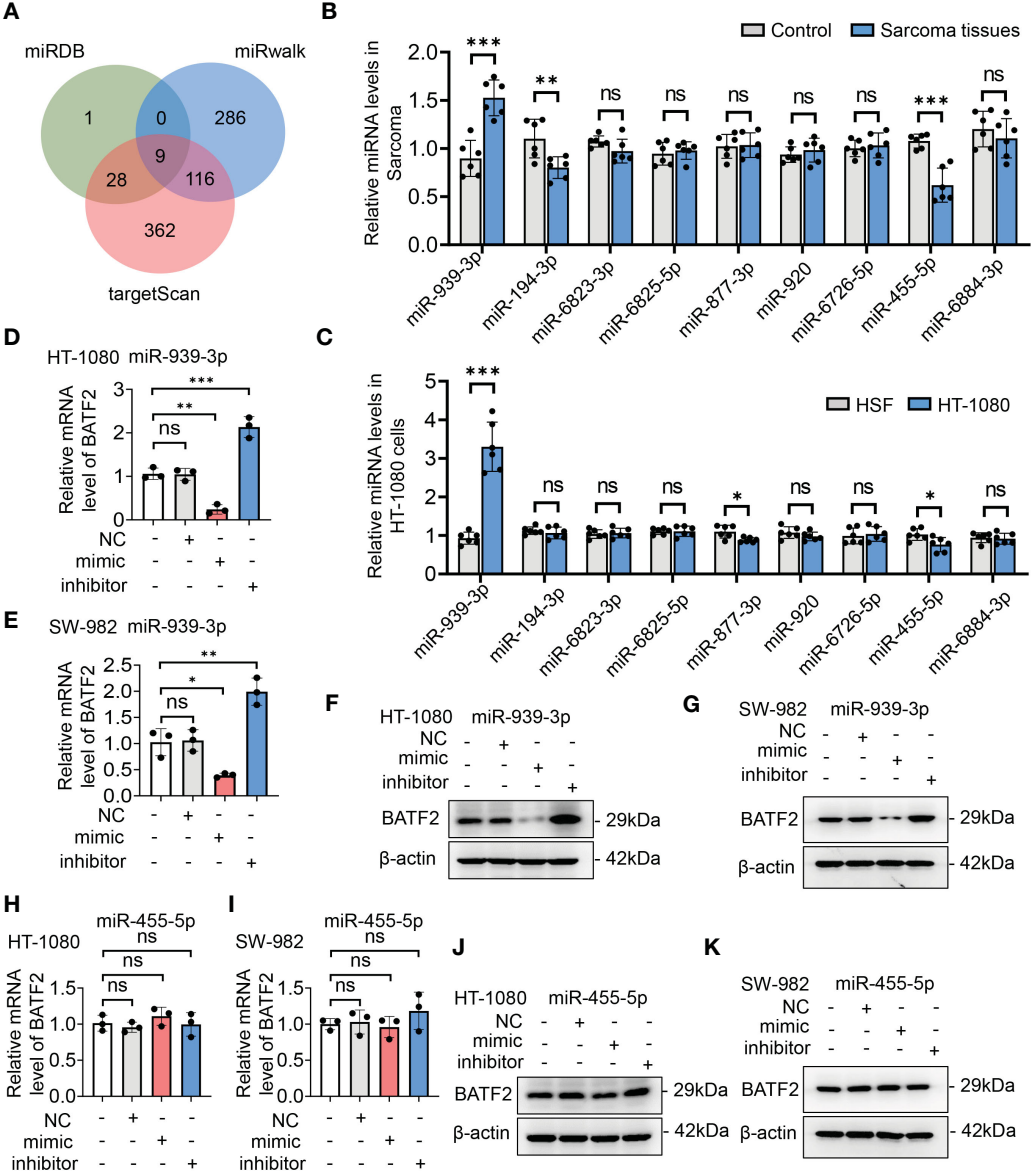
BATF2 expression was repressed by miR-939-3p in sarcoma. **(A)** miRNAs in the 3′-untranslated region (UTR) region of BATF2 were predicted by using miRDB, miRwalk and targetScan software programs, and 9 common miRNAs were found. **(B, C)** qPCR screening of the predicted 9 miRNAs in control and sarcoma tissues from sarcoma patients **(B)** and HSF and HT-1080 cells **(C)**. **(D–K)** qPCR and Western blot analysis of BATF2 expression in HT-1080 cells or SW-982 cells transfected with the mimic or inhibitor of miR-939-3p or miR-455-5p. ns: not significant. *P < 0.05, **P < 0.01, ***P < 0.001.

Since miRNAs play an extensive role in modulating gene expression in sarcoma (21, 22), we hypothesized that BATF2 might be regulated by miRNA. To test this hypothesis, we utilized miRDB, miRwalk and targetScan software programs to identify miRNAs that had potential binding sites in the 3′ UTR of BATF2 (**Figure 2A**). We then screened the predicted nine miRNAs using quantitative PCR (qPCR) in both sarcoma tissues (**Figure 2B**) and cell lines (**Figure 2C**), which showed that miR-939-3p and miR-455-5p were upregulated in sarcoma tissues and cells compared to controls. Further, BATF2 expression was reduced by miR-939-3p mimic and increased by miR-939-3p inhibitor in both HT-1080 and SW-982 cells (**Figures 2D–G**). However, neither the mimic nor the inhibitor of miR-455-5p could regulate BATF2 expression (**Figures 2H–K**), suggesting that BATF2 expression was repressed by miR-939-3p in sarcoma.

### 3.3 Upregulation of miR-939-3p associates with poor prognosis of sarcoma

**Figure 3:**
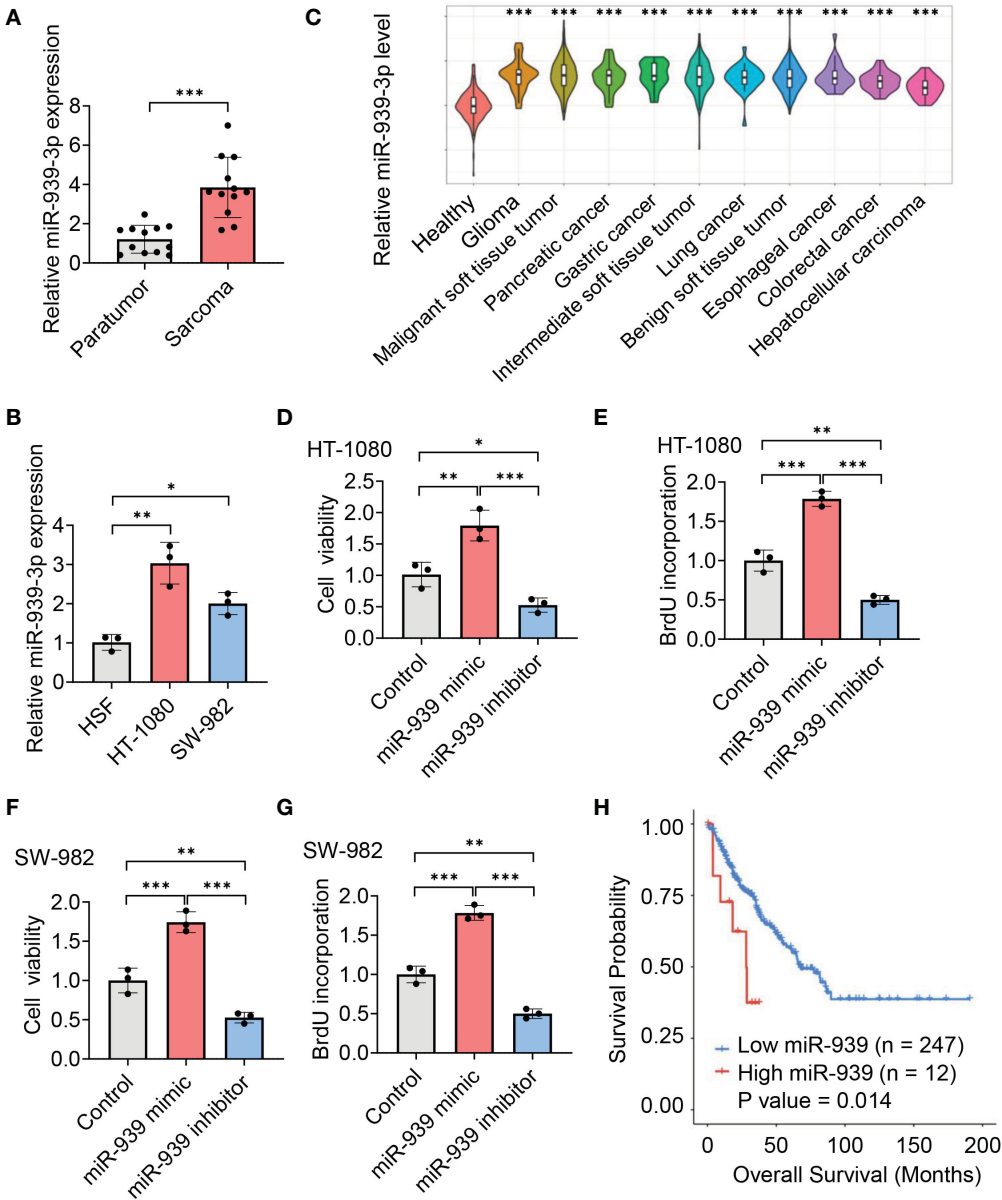
Upregulation of miR-939-3p associates with poor prognosis of sarcoma. **(A, B)** qPCR analysis of the expression of miR-939-3p in soft tissue sarcoma and pericarcinoma **(A)** and the human skin fibroblast cell line (HSF), human fibrosarcoma cell line (HT-1080) and synovial sarcoma cell line (SW-982) **(B)**. **(C)** Circulating miR-939-3p levels in healthy individuals (n = 275) and sarcoma patients with various histological subtypes, including glioma (n = 30), malignant soft tissue tumor (n = 402), pancreatic cancer (n = 30), gastric cancer (n = 30), intermediate soft tissue tumor (n = 144), lung cancer (n = 30), benign soft tissue tumor (n = 30), esophageal cancer (n = 30), colorectal cancer (n = 30) and hepatocellular carcinoma (n = 30), were analyzed by using a CancerMIRNome database. **(D–G)** Cell viabilities of HT-1080 or SW-982 cells transfected with control, miR-939-3p mimic or inhibitor separately for two days were analyzed by using cell counting kit 8 or BrdU ELISA kit. **(H)** Kaplan-Meier estimates of overall survival time based on miR-939 expression levels from 259 sarcoma patients by using a CancerMIRNome database. Hazard ratio (HR) = 2.69. *P < 0.05, **P < 0.01, ***P < 0.001.

To explore the role of miR-939-3p, we collected sarcoma tissues and observed an elevated expression of miR-939-3p compared to pericarcinoma tissues (**Figure 3A**). Similarly, sarcoma cell lines (HT-1080 and SW-982) also exhibited higher levels of miR-939-3p compared to a human skin fibroblast cell line (HSF) (**Figure 3B**). Moreover, circulating miR-939-3p levels were found to be upregulated in sarcoma patients with various histological subtypes, including glioma, malignant soft tissue tumor, pancreatic cancer, gastric cancer, intermediate soft tissue tumor, lung cancer, benign soft tissue tumor, esophageal cancer, colorectal cancer and hepatocellular carcinoma, compared to healthy individuals (**Figure 3C**).

Functional experiments further revealed that the viabilities of HT-1080 and SW-982 cells were enhanced by miR-939-3p mimic, while suppressed by miR-939-3p inhibitor, as evidenced by CCK-8 and BrdU incorporation assay (**Figures 3D–G**). Consistent with our hypothesis, Kaplan Meier survival analysis showed a negative correlation between miR-939-3p expression levels and the prognosis of sarcoma patients, as analyzed using both the CancerMIRNome database (*P* = 0.014, with a hazard ratio of 2.69) (**Figure 3H**) and UALCAN databases (*P* = 0.0036) (**Supplementary Figure S1**). These data revealed that miR-939-3p was significantly upregulated in sarcoma and was inversely associated with the prognosis of sarcoma patients.

### 3.4 miR-939-3p suppresses BATF2 expression via directly binding to its 3’ UTR

**Figure 4:**
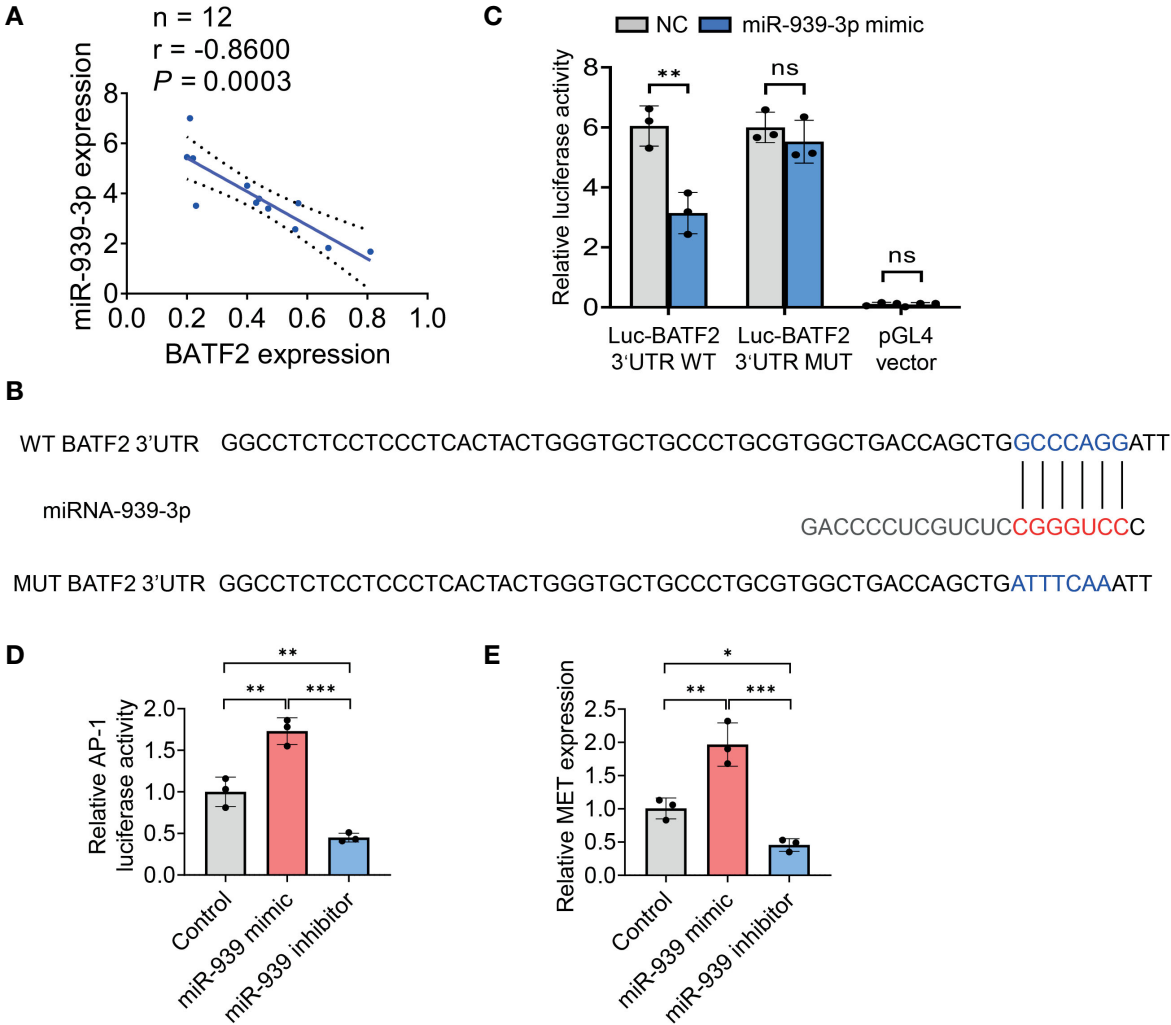
miR-939-3p suppresses BATF2 expression via binding to its 3’UTR region. **(A)** Statistical analysis of the negative correlation between miR-939-3p and BATF2 mRNA expression levels by using linear regression and Pearson correlation analysis. **(B)** Bioinformatic analysis of the binding sites of miR-939-3p in the 3’ UTR of BATF2 mRNA. **(C)** A luciferase reporter assay was performed to verify the binding of miR-939-3p in the 3’ UTR of BATF2 mRNA. The wild type (WT) 3’ UTR (GCCCAGG) was mutated into mutant 3’ UTR (ATTTCAA). ns: not significant. **P < 0.01. **(D)** pAP-1-Luc was co-transfected with Renilla into HT-1080 cells in combined with control or miR-939 mimic or inhibitor for luciferase reporter gene assay. pAP-1-Luc activity was normalized against Renilla activity. **(E)** qPCR analysis of MET expression in HT-1080 cells transfected with control or miR-939 mimic or inhibitor. *P < 0.05, **P < 0.01, ***P < 0.001.

Statistical analysis showed a significantly negative correlation between miR-939-3p and BATF2 expression levels in both sarcoma tissues (r = -0.8600, *P* = 0.0003) (**Figure 4A**) and colon cancer tissues (r = -0.7162, *P* = 0.0088) (**Supplementary Figure S2**), implying the involvement of a potential miR-939-3p/BATF2 signaling pathway in sarcoma. Based on the predicted binding sites of miR-939-3p in the 3’ UTR of BATF2 mRNA (**Figure 4B**), we postulated that miR-939-3p could suppress BATF2 expression through binding to its 3’ UTR. As shown in **Figure 4C**, luciferase reporter assays confirmed that overexpression of miR-939-3p suppressed the luciferase activity of wild-type (WT) recombinant BATF2 3’ UTR. As expected, miR-939-3p mimic had no effect on the fluorescence intensity in mutant BATF2 3’ UTR, where potential binding sites were mutated into ATTTCAA (**Figures 4B, C**). To validate the miR-939-3p/BATF2 axis, we further examined the downstream genes of BATF2 (13). Luciferase reporter gene assay and qPCR analysis showed that both AP-1 activity and MET proto-oncogene receptor tyrosine kinase (MET) expression were induced by miR-939-3p mimic, while repressed by miR-939-3p inhibitor (**Figures 4D, E**). These results demonstrated that miR-939-3p suppresses BATF2 expression through directly binding to its 3’ UTR.

### 3.5 Overexpression of BATF2 attenuates miR-939-3p-mediated sarcoma proliferation

**Figure 5:**
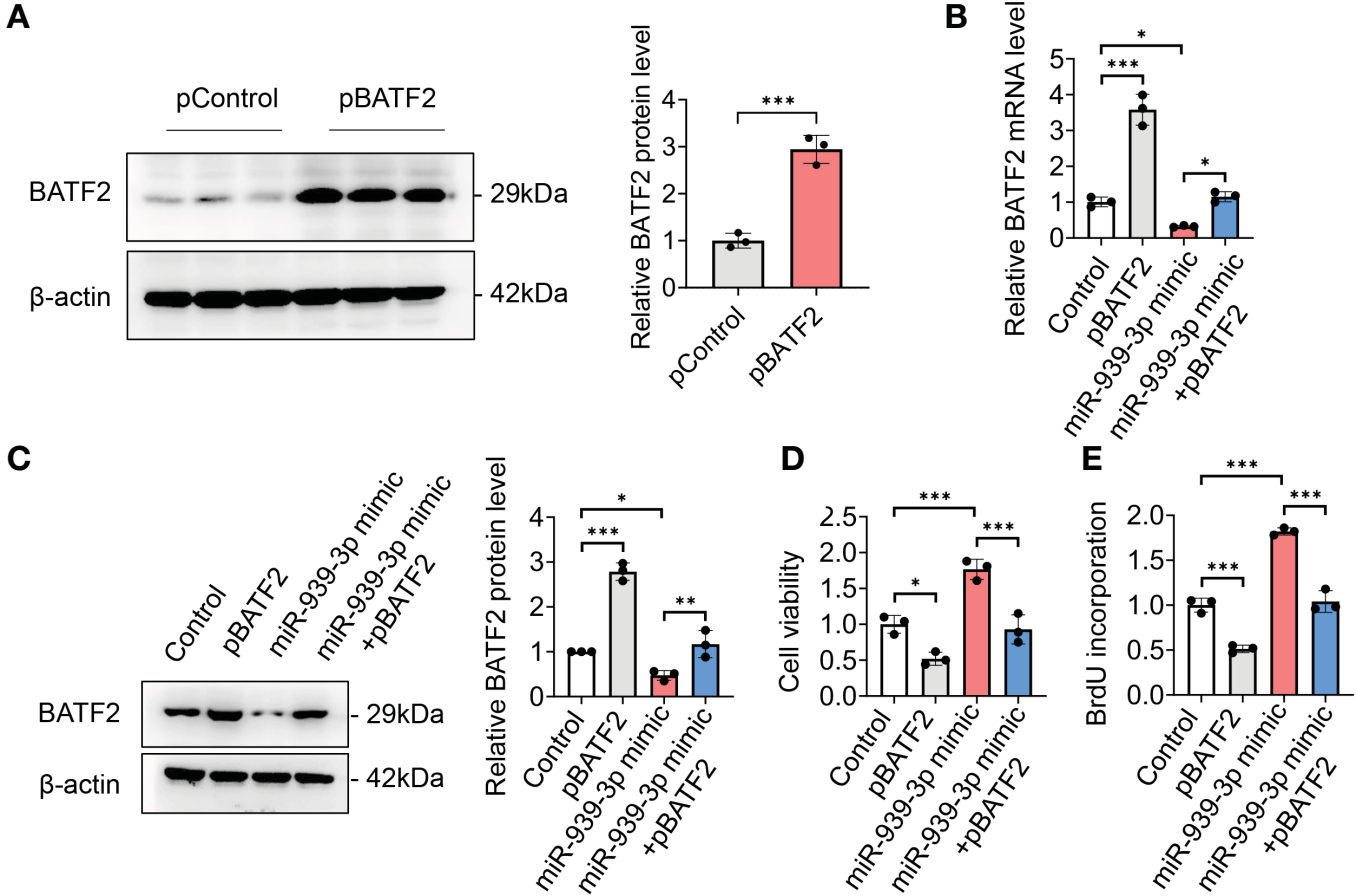
Overexpression of BATF2 attenuates miR-939-3p-mediated cancer proliferation. **(A)** Recombinant BATF2 plasmids (pBATF2) or control plasmids (pControl) were separately transfected into HT-1080 cells by using Lipofectamine 3000. **(B, C)** qPCR and Western blot analysis of BATF2 expression in HT-1080 cells transfected with pControl or pBATF2 treated with control or miR-939-3p mimic. **(D, E)** Cell viability of cells in **(B)** was analyzed by using CCK-8 or BrdU ELISA kit. ns: not significant, *P < 0.05, **P < 0.01, ***P < 0.001.

To investigate whether miR-939-3p-mediated regulation of BATF2 is involved in sarcoma proliferation, HT-1080 cells were transfected with BATF2 overexpression plasmids (pBATF2) or control plasmids (pControl) (**Figure 5A**). It was found that overexpression of BATF2 significantly alleviated the miR-939-3p-mediated suppression of BATF2 expression and promotion of sarcoma cell proliferation (**Figures 5B–E**), as well as cell migration and invasion (**Supplementary Figure S3**). Besides, miR-939-3p could neither further enhance sarcoma cell proliferation nor regulate AP-1 activity or MET expression, when BATF2 was knocked down by a large amount of siRNA in HT-1080 cells (**Supplementary Figures S4A–E**). These findings collectively suggest that miR-939-3p could induce sarcoma cell proliferation via inhibiting the expression of BATF2.

### 3.6 miR-939-3p enhances sarcoma growth *in vivo*

**Figure 6:**
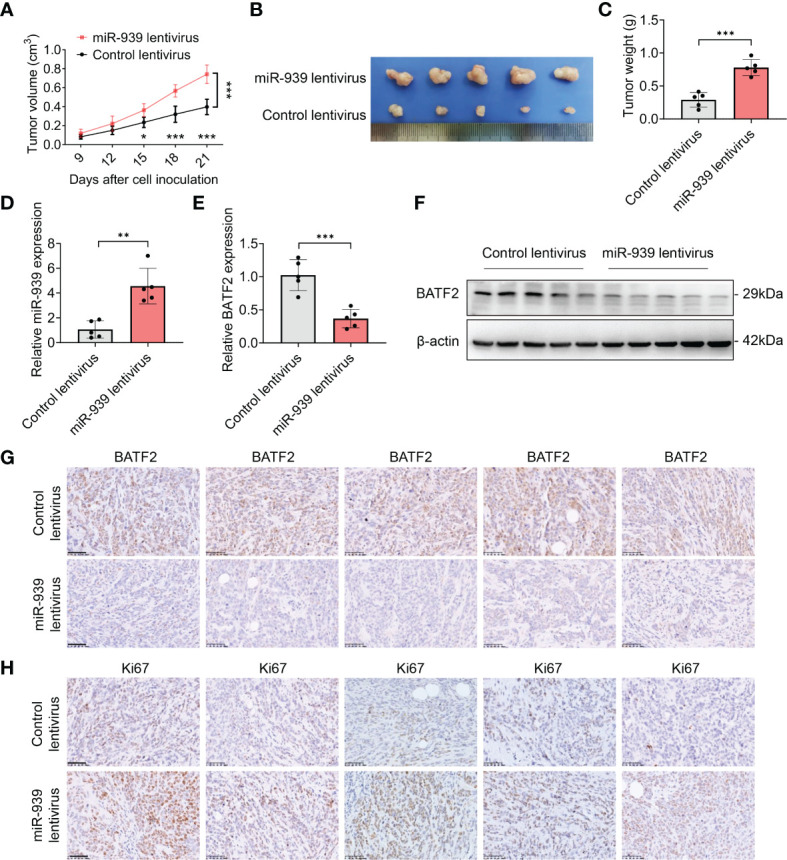
miR-939-3p enhances sarcoma growth *in vivo*. Xenograft nude mouse models were constructed by subcutaneously injecting 5 × 10^6^ HT-1080 cells stably infected with lentivirus expressing miR-939-3p or control separately (n = 5 per group). **(A)** The tumor volume was calculated every 3 days, and the mice were sacrificed 21 days after cell inoculation. **(B, C)** The xenografts were excised for the comparisons of tumor size and weight. **(D)** qPCR analysis of miR-939-3p expression in the xenografts. **(E, F)** qPCR and Western blot analyses of BATF2 expression in the xenografts. **(G, H)** Immunohistochemical analyses of the expressions levels of BATF2 and Ki67 in tumor xenografts. n = 5 per group. Data are expressed as mean ± SD. *P < 0.05, **P < 0.01, ***P < 0.001.

To further examine the role of miR-939-3p, *in vivo* experiments utilizing xenograft nude mouse models were conducted. HT-1080 cells stably infected with lentivirus expressing miR-939-3p or control were injected into the mice separately. Compared to the control mice, the miR-939-3p group exhibited larger tumor volumes and weights (**Figures 6A–C**). As anticipated, miR-939-3p expression was significantly higher in the xenografts of miR-939-3p group, whereas the expression levels of BATF2 mRNA and protein were much lower, compared to the control mice (**Figures 6D–G**), while immunohistochemical staining of Ki67, a biomarker of cell proliferation, showed that Ki67 expression was much higher in tumor xenografts from miR-939-treated mice than that in control mice (**Figure 6H**). These data revealed that miR-939-3p enhanced sarcoma growth *in vivo*.

## 4 Discussion

Sarcoma is a type of mesenchymal neoplasm exhibiting distinct prognosis, clinical behavior and treatment options (1, 28, 29). Sarcoma is regarded as a formidable type of malignancy to treat, as conventional chemotherapies have yielded frustratingly slow progress, while immunotherapy remains an investigational approach, making sarcoma one of the most difficult tumors to treat (4). Due to its rarity and histologic heterogeneity, the understanding of sarcoma biology is also relatively limited, compared with that of common epithelial cancers (1). Notably, the clinical behavior of sarcoma results from interactions at many levels, among which the oncogenes and tumor suppressor genes are crucial driving force for tumorigenesis and progression (8, 30). We and others have previously found that BATF2 is an important tumor suppressor in many cancers (9–18), although its role in sarcoma remains unknown. In this study, we found that BATF2 was downregulated in sarcoma tissues and cells, correlating with a poor prognosis of sarcoma patients. Further investigation revealed that BATF2 inhibited the proliferation of sarcoma cells, although the regulatory mechanism and its role in sarcoma remain unclear.

BATF2 belongs to the activator protein 1 (AP-1) family and inhibits AP-1 activity mainly through interacting with AP-1 via its bZIP domain, thereby leading to tumor repression (10–13). It has been reported that BATF2 is primarily expressed in normal cells but not in the corresponding tumor cells, while the upregulation of BATF2 has been shown to inhibit cancer cell proliferation, angiogenesis, invasion and metastasis (13, 14), underscoring its importance in cancer therapy. Because of the myriad of tumor suppressor functions of BATF2, it is imperative to elucidate the mechanisms underlying how BATF2 is regulated during tumorigenesis and development. We and others have recently explored the regulators of BATF2, including transcriptional factors, N6-methyladenosine modification and nuclear-cytoplasmic translocation (9, 10, 15). Our previous studies have reported glucocorticoid receptor, a transcriptional factor, which suppresses lymphoma cells through inducing BATF2 expression (10). We have also found that repressing the nuclear export of BATF2 by mutating its nuclear export sequence and suppressing the expression of chromosome region maintenance 1 (CRM1) might serve as potential therapeutic approaches for patients with colorectal cancer (9). Nevertheless, these studies have still not clarified the reason for the downregulation of BATF2, especially in sarcomas.

In this study, the bioinformatic analysis and experimental verification identified miR-939-3p as a candidate regulator of BATF2 in sarcoma tissues and cell lines, and upregulation of miR-939-3p was associated with a poor prognosis of sarcoma patients, suggesting its role as a potent prognostic biomarker and therapeutic target during sarcoma progression. As reported, miRNAs play a vital role in gene regulatory networks, and have gradually been demonstrated as promising diagnostic and prognostic biomarkers in human cancers, as well as potential targets for cancer therapy (22, 23, 31). More importantly, accumulated studies have established the therapeutic potential of miRNA mimics or antagomirs against cancer (32–36). Specifically, miR-939-3p is involved in the pathological processes of tumorigenesis and progression (37–39). In ovarian cancer, miR-939 was demonstrated to promote tumor proliferation by repressing the expression of adenomatous polyposis coli 2 (APC2) (37). In hepatocellular carcinoma, miR-939-3p induced epithelial-mesenchymal transition via targeting estrogen receptor 1 (ESR1) (38). Besides, miR-939-3p was identified as a potential independent prognostic marker in lung cancer (39). Although miR-939-3p has been studied in several cancers, its contribution to sarcoma proliferation had not been investigated. This work demonstrated for the first time that upregulation of miR-939-3p promoted sarcoma cell proliferation *in vitro* and enhanced tumor growth *in vivo*. Notably, clinical survival analysis revealed a negative correlation between miR-939-3p and BATF2 expression levels, and subsequent mechanistic studies illustrated that miR-939-3p suppressed BATF2 expression via binding to its 3’ UTR region. It has also been reported that miR-939 could induce mRNA degradation of its target gene (40), further supporting our results. These findings may shed new light on the oncogenic role of miR-939-3p targeting BATF2 in sarcomas.

Moreover, this study also found that circulating miR-939-3p levels were higher in tumor patients than that in healthy individuals. Due to their stable existence in circulation and enormous potential as non-invasive early detection biomarkers for cancer (22, 23), exploring the role of circulating miR-939-3p as a biomarker for sarcomas is also an interesting topic. Besides, it has been reported that miRNA-765 mediated multidrug resistance (MDR) via targeting BATF2 in gastric cancer cells (19). To investigate the roles of miRNA in chemo-resistance, a previous report have examined the miRNA expression profiles on 4 responder and 4 non-responder colon cancer to chemotherapy (41). The miRNA profiling data indicated that miR-939 was one of the most upregulated miRNAs in responder, compared with non-responder. However, subsequent screening and validation experiments excluded miR-939, since there was no significant difference in the expression level of miR-939 between non-responder and responder colon cancer patients (41). Therefore, it would be also interesting to explore the association between miR-939-3p and MDR in the future studies.

**Figure 7:**
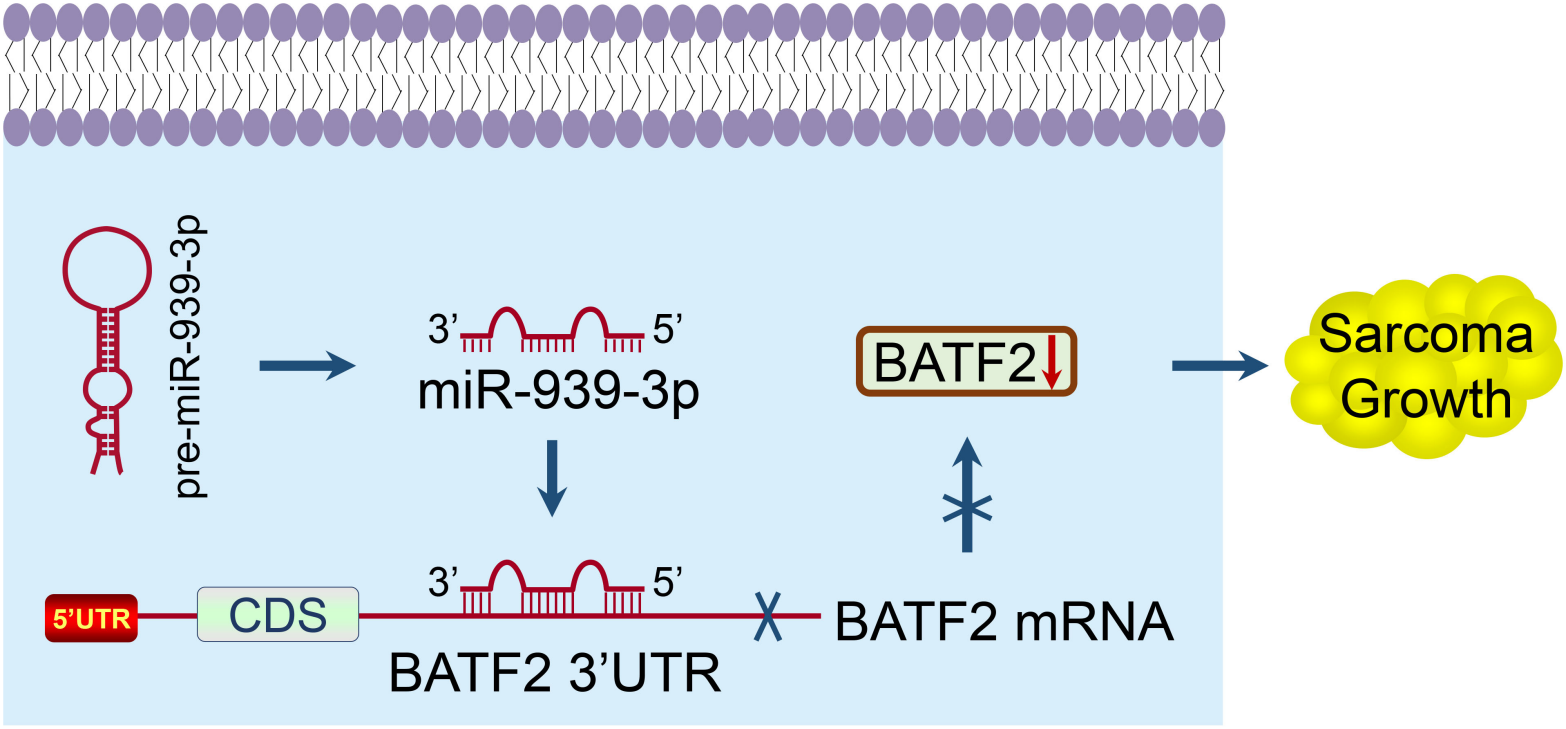
A proposed model elucidating the mechanism of miR-939-3p-induced sarcoma proliferation and poor prognosis through suppressing BATF2 via directly binding to its 3’ UTR.

In summary, this study identified BATF2 as the target gene of miR-939-3p in sarcoma, and revealed that both upregulated miR-939-3p and downregulated BATF2 expression levels were significantly associated with a poor prognosis of sarcoma patients. Further mechanistic studies demonstrated that miR-939-3p promoted sarcoma cell proliferation via binding to the 3’ UTR of BATF2 (**Figure 7**). These findings suggest that suppressing miR-939-3p or inducing BATF2 expression may represent a new therapeutic strategy for sarcoma treatment, which may provide novel insights for the development of potent therapeutic approaches for sarcoma patients.

## Data availability statement

The original contributions presented in the study are included in the article/**Supplementary Material**. Further inquiries can be directed to the corresponding authors.

## Ethics statement

The studies involving humans were approved by People’s Hospital of Xishui County. The studies were conducted in accordance with the local legislation and institutional requirements. The participants provided their written informed consent to participate in this study. The animal study was approved by Animal Welfare and Ethics Committee of Army Medical University. The study was conducted in accordance with the local legislation and institutional requirements.

## Author contributions

WX: Data curation, Formal analysis, Investigation, Methodology, Resources, Writing – original draft. YH: Funding acquisition, Methodology, Software, Validation, Writing – review & editing. ZL: Software, Supervision, Validation, Visualization, Writing – review & editing. JZ: Conceptualization, Funding acquisition, Project administration, Supervision, Writing – review & editing.
