# Supplementary figures and images for: miR-939-3p induces sarcoma proliferation and poor prognosis via suppressing BATF2
Source: Front Oncol. 2024 Feb 14;14:1346531. doi: 10.3389/fonc.2024.1346531 (PMC10899471; doi:10.3389/fonc.2024.1346531)

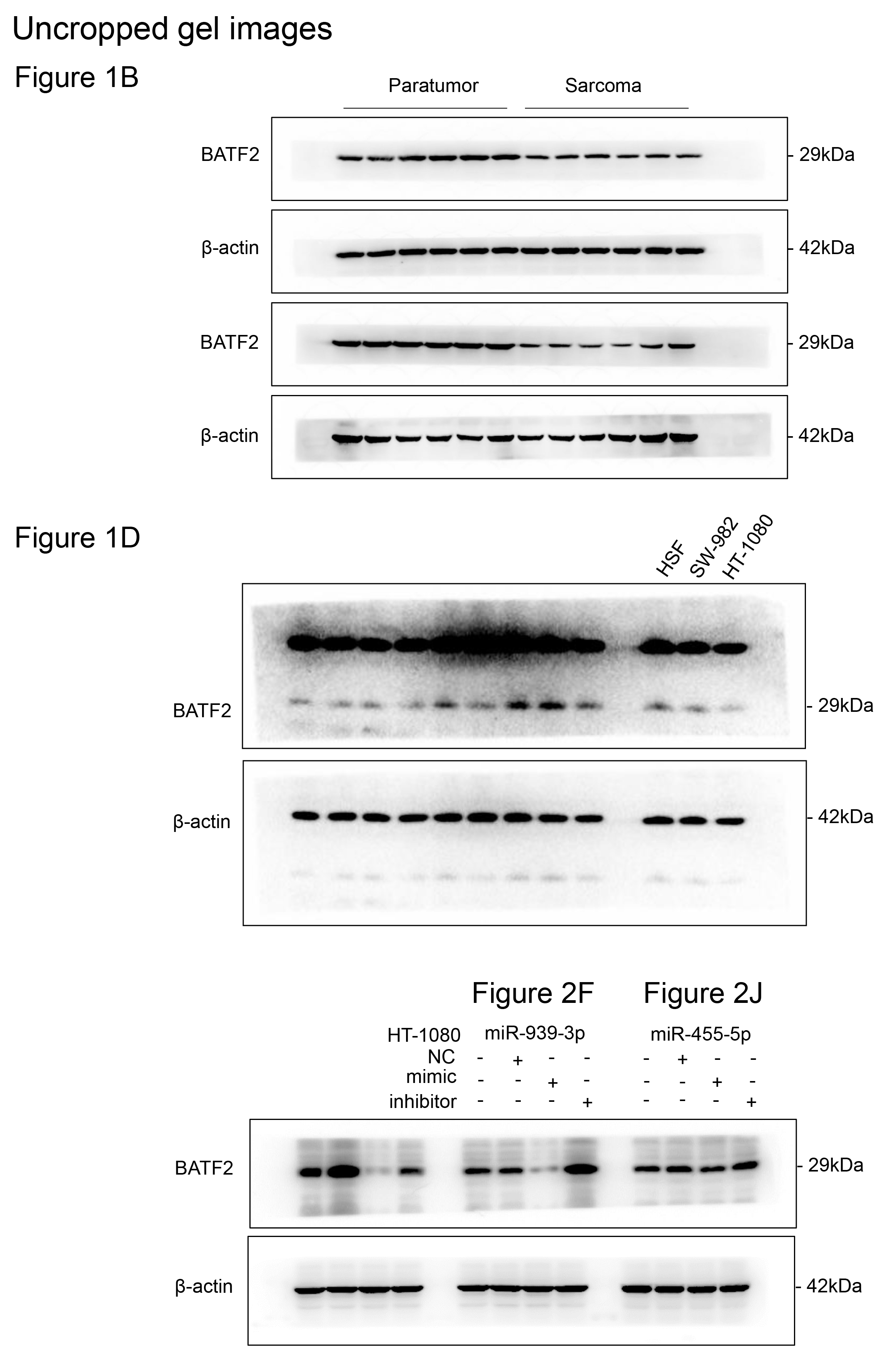

Supplement: Supplementary file 1 [file DataSheet_1.zip › Raw data 20240124修回/Uncropped gel images 1.tif]

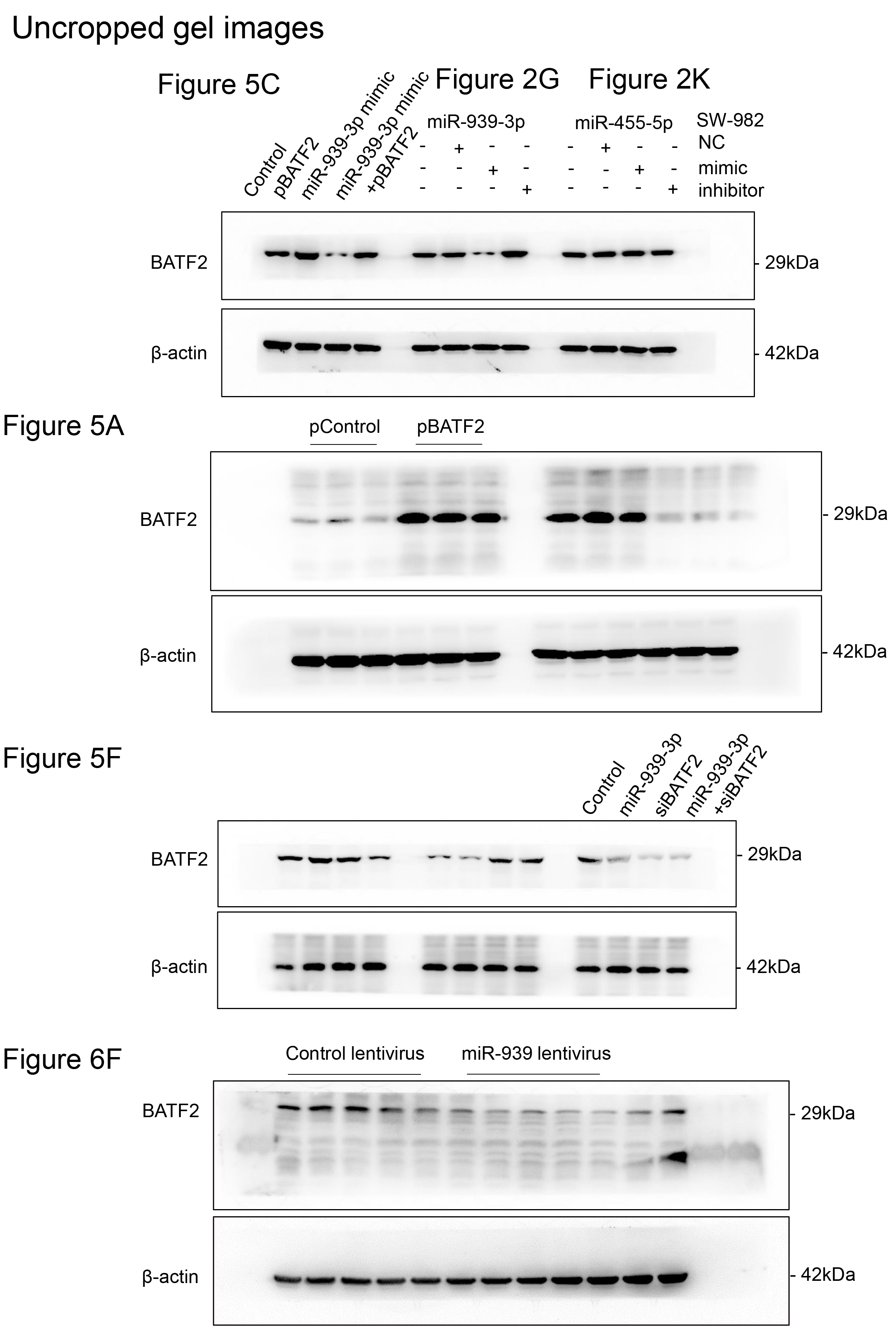

Supplement: Supplementary file 1 [file DataSheet_1.zip › Raw data 20240124修回/Uncropped gel images 2.tif]
