# Supplementary material for: miR-939-3p induces sarcoma proliferation and poor prognosis via suppressing BATF2
Source: Front Oncol. 2024 Feb 14;14:1346531. doi: 10.3389/fonc.2024.1346531 (PMC10899471; doi:10.3389/fonc.2024.1346531)
Supplement: Supplementary file 2 [file DataSheet_2.docx]

Supplementary Material

# Supplementary methods

**UALCAN analysis**

UALCAN ([1](#_ENREF_1), [2](#_ENREF_2)) is an online data-mining resource that allows analysis of gene expression profiles in various cancer types based on publicly available cancer OMICS data, including TCGA, CBTTC and CPTAC. It also provides patient survival information for miRNAs and identifies potential tumor biomarkers.

**Wound healing assay**

Cell migration was evaluated by wound healing assay in HT-1080 cells transfected with miR-939-3p mimic in combined with BATF2 overexpression plasmids or negative control at 0 h and 24 h. After the scratch using a 200 μL pipette tip, cells in 12-well plates were washed with PBS and then photographed at 0 h and 24 h later by using an inverted microscope.

**Cell invasion assay**

Cell invasion assay was performed as previously described ([3](#_ENREF_3)). The 24-well Transwell plates and Matrigel basement membrane matrix were obtained from Corning (Corning, NY, USA).

**Supplementary references**

1. Chandrashekar DS, Karthikeyan SK, Korla PK, Patel H, Shovon AR, Athar M, et al. UALCAN: An update to the integrated cancer data analysis platform. Neoplasia. 2022;25:18-27.

2. Chen F, Chandrashekar DS, Varambally S, Creighton CJ. Pan-cancer molecular subtypes revealed by mass-spectrometry-based proteomic characterization of more than 500 human cancers. Nature communications. 2019;10(1):5679.

3. Wang RY, Chen L, Chen HY, Hu L, Li L, Sun HY, et al. MUC15 inhibits dimerization of EGFR and PI3K-AKT signaling and is associated with aggressive hepatocellular carcinomas in patients. Gastroenterology. 2013;145(6):1436-48.e1-12.

# Supplementary Figures and Tables

## Supplementary Figures

**
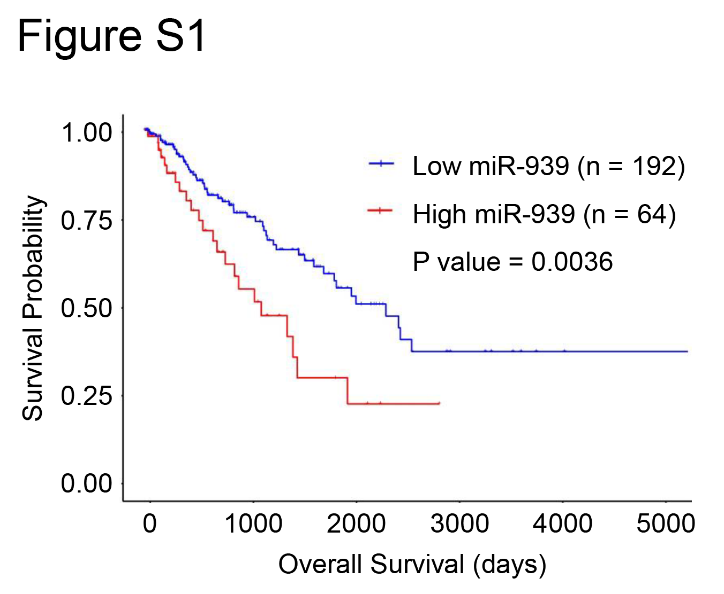
**

**Supplementary Figure 1.** **miR-939-3p correlates with poor prognosis of sarcoma.**

Kaplan-Meier estimates of overall survival time based on miRNA-939 expression levels from 256 sarcoma patients by using a UALCAN database. *P* =0.0036.


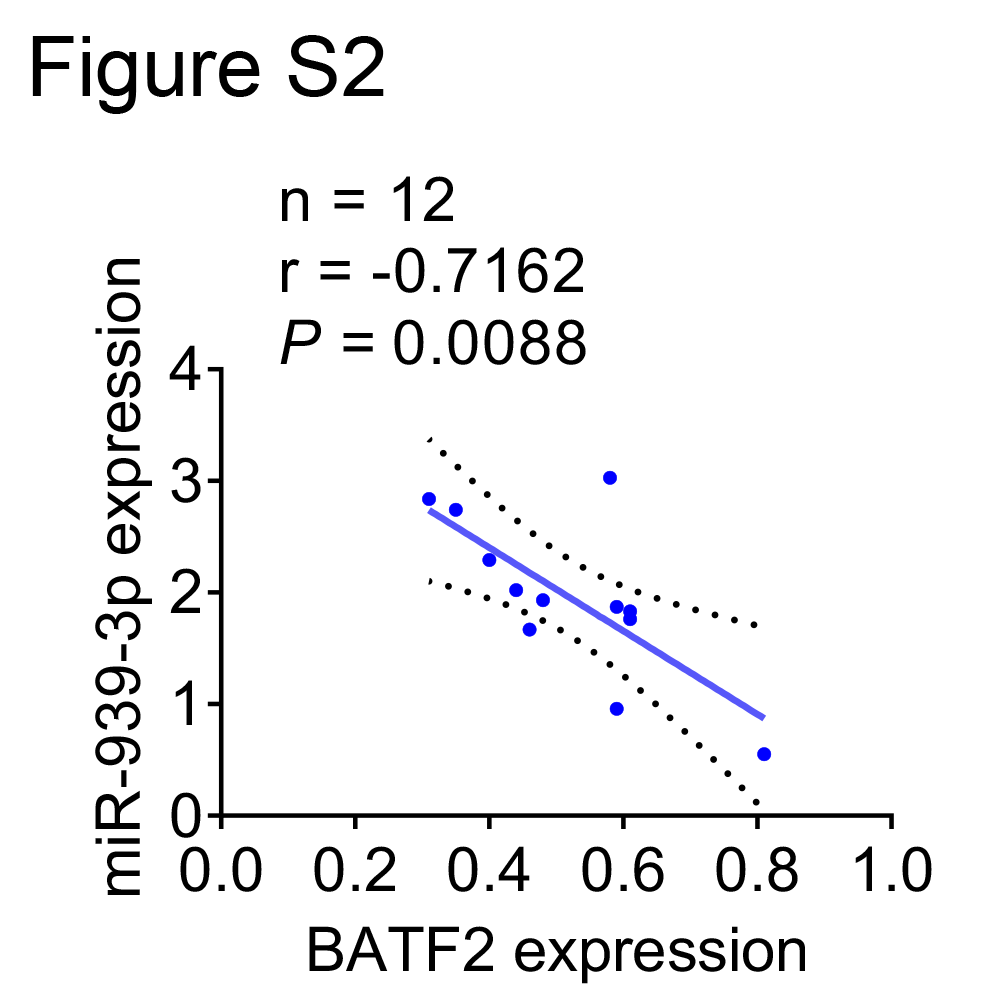


**Supplementary Figure 2.** **miR-939-3p negatively associates with BATF2 expression in CRC.**

Statistical analysis of the negative correlation between miR-939-3p and BATF2 mRNA expression levels in 12 CRC tissues by using linear regression and Pearson correlation analysis. *P* =0.0088.


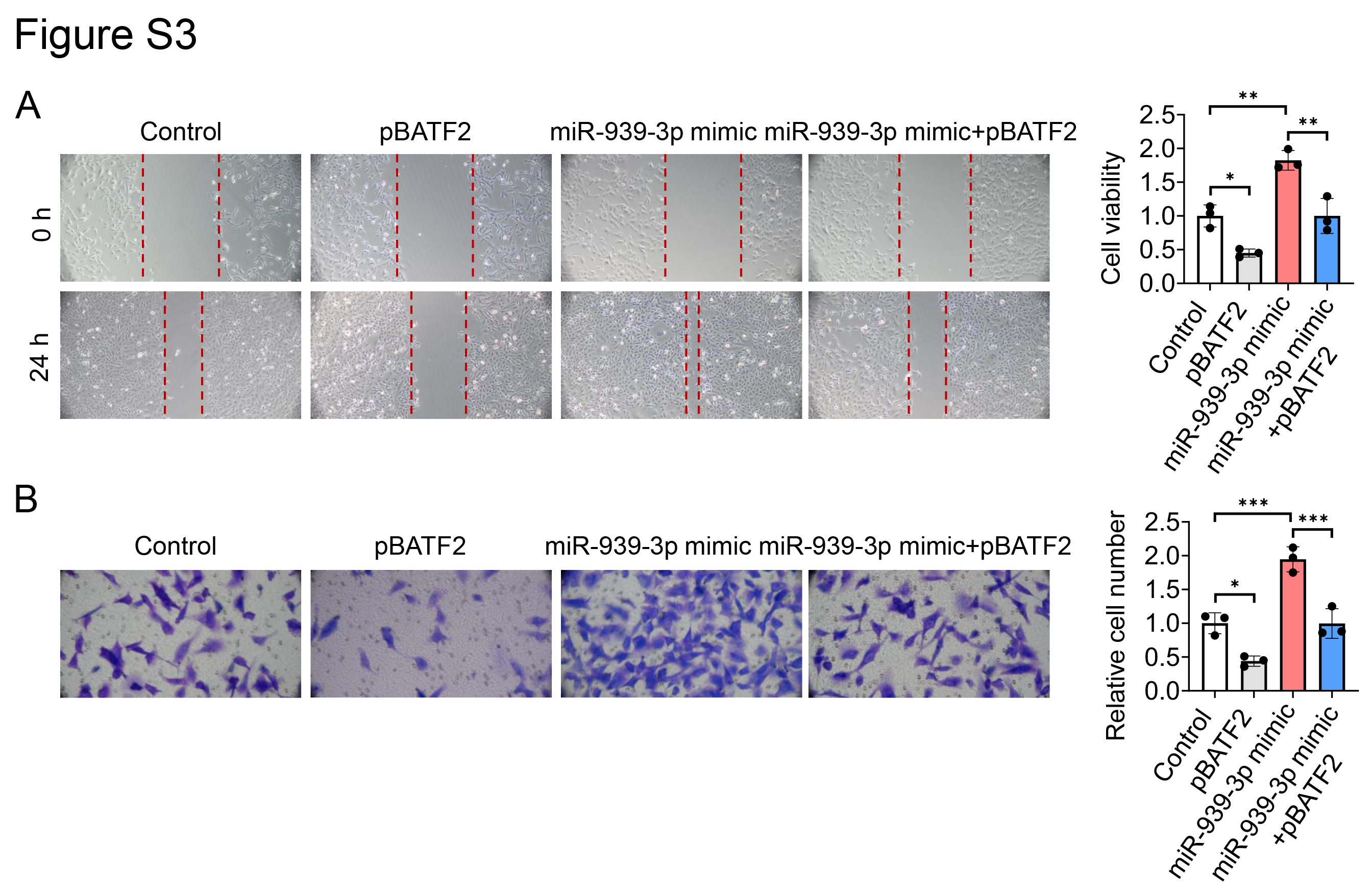


**Supplementary Figure 3.** **Overexpression of BATF2 ameliorates miR-939-3p-mediated sarcoma cell migration and invasion.**

(A) The cell migration was evaluated by wound healing assay in HT-1080 cells transiently transfected with miR-939-3p mimic in combined with BATF2 overexpression plasmids or negative control at 0 h and 24 h. (B) HT-1080 cells were transfected with miR-939-3p mimic in combined with BATF2 overexpression plasmids or negative control, and then subjected to the Matrigel invasion assay. **P <* 0.05, ***P* < 0.01, ****P* < 0.001.


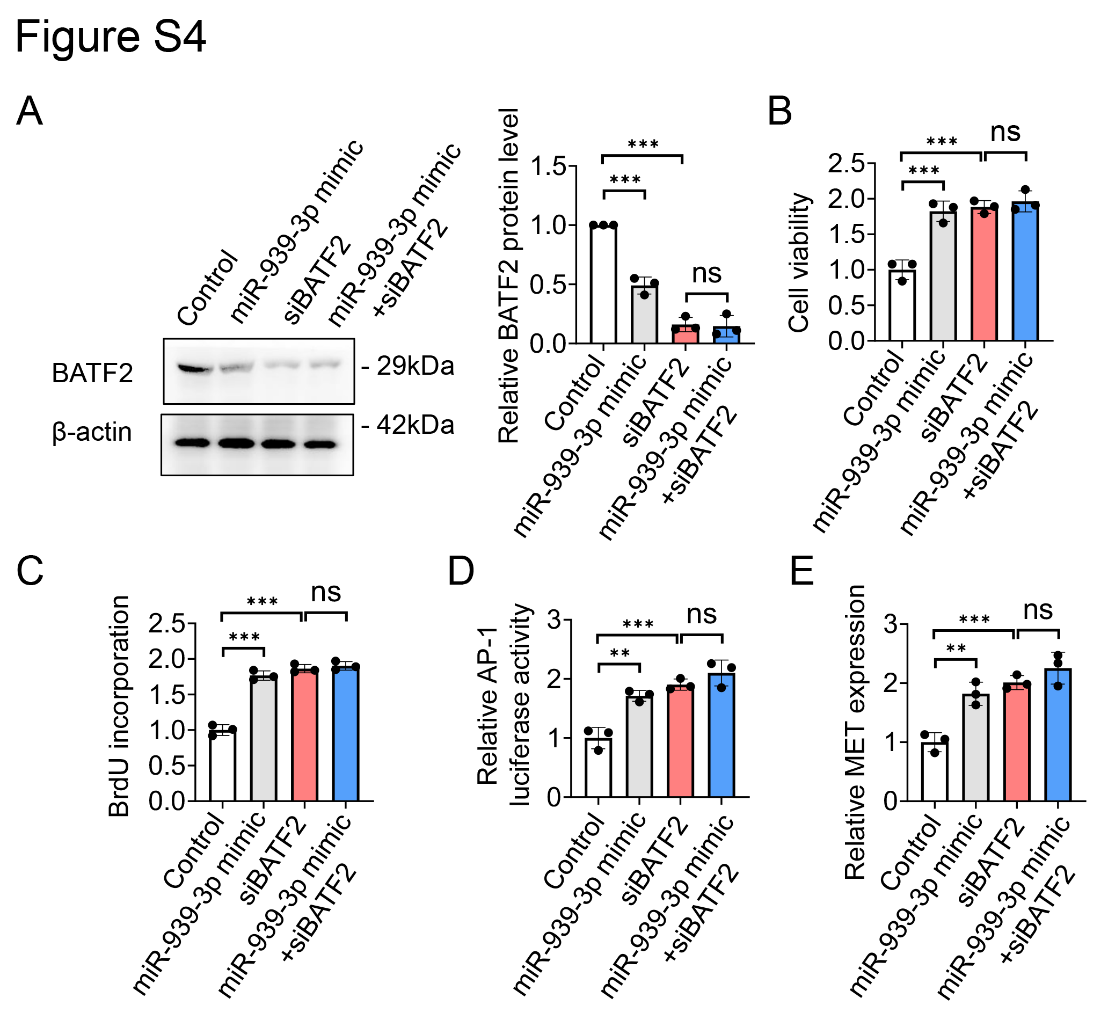


**Supplementary Figure 4.** **miR-939-3p could neither further enhance sarcoma cell proliferation nor inhibit BATF2 or its target gene expression when BATF2 was significantly knocked down.**

(A) Western blot analysis of BATF2 expression in HT-1080 cells transfected with negative control or siRNAs targeting BATF2 (siBATF2) and then control or miR-939-3p mimic. (B and C) Viabilities of cells in (A) was analyzed by using CCK-8 or BrdU ELISA kit. (D) pAP-1-Luc was co-transfected with Renilla into HT-1080 cells in combined with siBATF2 and control or miR-939-3p mimic for luciferase reporter gene assay. pAP-1-Luc activity was normalized against Renilla activity. (E) qPCR analysis of MET expression cells in (D). ns: not significant, ***P* < 0.01, ****P* < 0.001.

## Supplementary Tables

**Supplementary Table 1.** The primer sets for mRNAs

| Gene | Sequences |
| --- | --- |
| *BATF2* | Forward: 5'- CCTGCACCAGCAGCACGAGTC -3' |
|  | Reverse: 5'- GCTCCCGGCAGCCATGTTGT -3' |
| *β-actin* | Forward: 5'- GTGAAGGTGACAGCAGTCGGTT -3' |
|  | Reverse: 5'- GAAGTGGGGTGGCTTTTAGGA -3' |

**Supplementary Table 2.** The primer sets for miRNAs

| miRNA | Primers |
| --- | --- |
| *miR-939-3p* | Forward: 5'- GACCCCTCGTCTCCGGGTCCC-3' |
| *miR-194-3p* | Forward: 5'- CCAGTGGGGCTGCTGTTATCTG -3' |
| *miR-6823-3p* | Forward: 5'- TGAGCCTCTCCTTCCCTCCAG -3' |
| *miR-6825-5p* | Forward: 5'- TGGGGAGGTGTGGAGTCAGCAT -3' |
| *miR-877-3p* | Forward: 5'- TCCTCTTCTCCCTCCTCCCAG -3' |
| *miR-920* | Forward: 5'- GGGGAGCTGTGGAAGCAGTA-3' |
| *miR-6726-5p* | Forward: 5'- CGGGAGCTGGGGTCTGCAGGT-3' |
| *miR-455-5p* | Forward: 5'- TATGTGCCTTTGGACTACATCG -3' |
| *miR-6884-3p* | Forward: 5'- CCCATCACCTTTCCGTCTCCCCT-3' |
| *Reverse* | Reverse: 5'-GCTGTCAACGATACGCTACGTAACG-3' |
| *U6* | Forward: 5'-CTCGCTTCGGCAGCACA-3' |
|  | Reverse: 5'-AACGCTTCACGAATTTGCGT-3' |

**Supplementary Table 3.** The mimics and inhibitors for miRNAs

| miRNA | Sequences |
| --- | --- |
| *miR-939-3p* mimics | Forward: 5'-CCCUGCCCCUCUGCUCCCCAG-3' |
|  | Reverse: 5'-CUGGGGAGCAGAGGCCCAGGG-3' |
| *miR-455-5p* mimics | Forward: 5'-UAUGUCCCUUUGGACUACAUCG-3' |
|  | Reverse: 5'-CGAUGUAGUCCAAAGGCACAUA-3' |
| *NC* mimics | Forward: 5'-UCACAACCUCCUAGAAAGAGUAGA-3' |
|  | Reverse: 5'-UCUACUCUUUCUAGGAGGUUGUGA-3' |
| *miR-939-3p* inhibitor | Reverse: 5'-CUGGGGAGCAGAGGCCCAGGG-3' |
| *miR-455-5p* inhibitor | Reverse: 5'-CGAUGUAGUCCAAAGGCACAUA-3' |
| *NC* inhibitor | Reverse: 5'-UCUACUCUUUCUAGGAGGUUGUGA-3' |

**Supplementary Table 4.** The primer sets for reporter plasmids construction^*^

| Order | Sequences |
| --- | --- |
| Forward | 5'- ACCTGAGCTCGCTAGCCTCGAGCCT GGTCTTCGGAGCTGGGTTGGCCCCTT -3' |
| Reverse | 5'- ACAGTACCGGATTGCCAAGCTTTTAAA GATTGCAAAACACTTTATTTCCATCACA -3' |
| ^*^ Restriction enzymes: XhoI and HindIII, Vector: pGL4. | |
